# GDNF triggers proliferation of rat C6 glioma cells via the NF-κB/CXCL1 signaling pathway

**DOI:** 10.1371/journal.pone.0289071

**Published:** 2023-08-18

**Authors:** Yue Wang, Yue Wu, Li Li, Jin Gao, Dian Shuai Gao, Shen Sun

**Affiliations:** 1 National Demonstration Center for Experimental Basic Medical Science Education, Xuzhou Medical University, Xuzhou, Jiangsu, China; 2 Department of Histology and Embryology, Xuzhou Medical University, Xuzhou, Jiangsu, China; 3 Department of Neurobiology and Anatomy, Xuzhou Key Laboratory of Neurobiology, Jiangsu Key Laboratory of New Drug Research and Clinical Pharmacy, Xuzhou Medical University, Xuzhou, Jiangsu, China; 4 Department of Pathophysiology, Xuzhou Medical University, Xuzhou, Jiangsu, China; 5 Department of Cell Biology, Xuzhou Medical University, Xuzhou, Jiangsu, China; Northwest University, UNITED STATES

## Abstract

Glioblastoma multiforme (GBM) is the most common primary malignant brain tumor that is characterized by its high proliferative and migratory potential, leading to a high invasiveness of this tumor type. However, the underlying mechanism of GBM proliferation and migration has not been fully elucidated. In this study, at first, we used RNA-seq together with bioinformatics technology to screen for C-X-C motif ligand 1 (CXCL1) as a proliferation-related gene. And exogenous glial cell line-derived neurotrophic factor (GDNF) induced proliferation and up-regulated the level of CXCL1 in rat C6 glioma cells determined by sqPCR and ELISA. Then, we manipulated the CXCL1 expression by using a lentiviral vector (CXCL1-RNAi) approach. By this, the proliferation of C6 cells was decreased, suggesting that CXCL1 plays a key role in proliferation in these cells. We hypothesized that exogenous GDNF promoted NF-κB nuclear translocation and therefore, analyzed the interaction of CXCL1 with NF-κB by Western Blot and immunofluorescence. Additionally, we used BAY 11–7082, a phosphorylation inhibitor of NF-κB, to elucidate NF-κB mediated the effect of GDNF on CXCL1. These results demonstrated that GDNF enhanced the proliferation of rat C6 glioma cells through activating the NF-κB/CXCL1 signaling pathway. In summary, these studies not only revealed the mechanism of action of exogenous GDNF in promoting the proliferation of C6 glioma cells but may also provide a new biological target for the treatment of malignant glioma.

## Introduction

Glioblastoma (GBM) is the most common primary malignant brain tumor, characterized by a high invasiveness [1]. Like other tumors, GBM cells display an increased proliferation and migration potential that can eventually lead to the death of the patients [2, 3]. However, the underlying mechanism of this cellular behavior has not been fully elucidated. Although the conventional treatment has some effect on glioma patients in early stages, the treatment effect of glioma patients in advanced stages is still very poor, mainly because glioma is prone to relapse and displays resistance to radio- and chemotherapy [4, 5]. It has been proved that many oncogenes or tumor suppressors are dysregulated in glioma, which lead to a malignant progression of the disease [4, 6, 7]. Therefore, studying the molecular mechanism of glioma development is conducive to exploring effective therapeutic strategies [4].

GDNF is a member of the transforming growth factor beta (TGF-beta) superfamily, which was first isolated and purified in 1993 [8]. The protein was previously considered to be an important differentiating factor with specific physiological roles in development and survival. Recent studies have shown that the tumorigenesis of glioma is associated with the abnormal expression of many cytokines, including GDNF [9–11]. Wiesenhofer and colleagues found an abnormal elevation of GDNF expression in primary glioma tissues and multiple glioma cell lines [12]. It is noteworthy that this increase was positively correlated with the pathological grade of the tumors [12]. Numerous studies suggest that exogenous GDNF plays an important role in proliferation and migration [11, 13, 14]. Moreover, GDNF plays a regulatory role in glioma progression and is part of a complex multi-molecule network [15]. However, up to now, it is unclear how GDNF influences this multi-molecular network to promote migration and proliferation of glioma cells.

CXCL1, also known as GRO-α (growth related oncogene-α), is a proangiogenic and chemotactic factor, which mediates neutrophil recruitment by binding to CXCR2 receptors. Recent studies indicated that the proliferation and migration of cancer cells can be linked to some chemokines of the tumor microenvironment [16, 17]. These molecules are secreted by tumor cells or other cell types present in the tumor microenvironment and that regulate the tumor immune response, angiogenesis, and proliferation [18]. In addition, the production of CXCL1 has also been shown to be upregulated in glioma tissues where it mediates the proliferation of glial progenitor cells during neurodevelopment and contributes to glioma formation [19]. However, the role of CXCL1 in the proliferation of glioma cells and its underlying mechanism remain unclear.

In this study, we found CXCL1 was a proliferation-related gene by RNA-seq together with bioinformatics technology, and exogenous GDNF up-regulated the level of CXCL1 in rat C6 glioma cells determined by sqPCR and ELISA. Then, we suggested that CXCL1 plays a key role in proliferation in these cells by CXCL1-RNAi approach. Furthermore, we demonstrated exogenous GDNF promoted NF-κB nuclear translocation and the interaction of CXCL1 with NF-κB, and NF-κB mediated the effect of GDNF on CXCL1 by Western Blot and immunofluorescence. This study not only reveals the mechanism underlying the promotion of C6 cell proliferation after exogenous GDNF administration but also provides a new biological target for the treatment of malignant glioma.

## Materials and methods

### Cells and culture conditions

The rat C6 glioma cell line was obtained from the Chinese Academy of Sciences (Shanghai, China). The cells were cultured in F12/DMEM medium supplemented with 10% FBS for routine culture and with 0.1% FBS for hunger culture. The C6 cells were grown in 10 cm dishes and subjected to exogenous GDNF. Untreated C6 cells were used as a control. The AST cells were obtained from our laboratory at Xuzhou Medical University.

### Antibodies and reagents

Antibodies were used at the following dilutions: Monoclonal mouse anti-β-catenin (BD610153; BD Biosciences, USA), 1:500; polyclonal rabbit anti-CXCL1 (GTX74108; GeneTex, USA), 1:2000; polyclonal rabbit anti-NF-κB (Cell Signaling Technology, USA), 1:2000; monoclonal mouse anti-β-actin (sc47778; Santa cruz, USA), 1:1000; polyclonal rabbit anti-Histone H3 (BS1660; Bioworld, USA), 1:500; IRDye 800CW goat (polyclonal) anti-rabbit IgG (926–32211; LI-COR, USA), 1:15000; IRDye 800CW goat (polyclonal) anti-mouse IgG (92632210; LI-COR, USA), 1:15000. BAY 11–7082 (S2913, Selleck, China).

### RNA-seq

C6 cells were cultured and treated with GDNF for 0, 0.5, 1, and 24 h, respectively and total RNA was extracted from the four sample groups using Trizol reagent. All experiments were run in triplicate. After passing the electrophoretic quality inspection, performed on an Agilent 2100 Bioanalyzer (Agilent Technologies, Santa Clara, CA, US), total RNA was purified with a RNA clean XP kit and RNase free DNase set. Bidirectional RNA-Seq of the four samples were performed with an Illumina hiseq2500 high-throughput sequencer.

### Screening of differentially expressed genes DEGs

The reads were converted into the number of reads per 1000 bases compared to exons in the reads per 1 million comparisons and the expression amount was standardized to calculate the gene’s fold change (FC). At the same time, the DEGs of different samples were analyzed by edger and the threshold value of *P* was determined by controlling the false discovery rate (FDR). The obtained *P* value was corrected by a multiple hypothesis test and the corrected *P* value was designated as Q value. Screening conditions for DEGs: Q value ≤ 0.05 and fold change ≥ 2 [20].

### Heat map and Venn map showed the distribution of DEGs

In this study, we used the heatmap3 package of R language for data visualization [21]. Additionally, Venny’s online software was used to draw a Venn graph. (http://bioinfogp.cnb.csic.es/tools/venny/index.html)

### Enrichment analysis of Go terminology and KEGG pathway

We used the David tool to perform GO and KEGG pathway enrichment analysis on the DEPs information. *P* < 0.05 was considered as being statistically significantly different [22].

### PPI network integration and module analysis

In this study, we used the STRTING tool to construct a PPI network (medium confidence: 0.400) for DEPs, GDNF, and CXCL1 that were both rising in cytoplasm and nucleus, and thereafter used the Cytoscape software for further module analysis [23].

### Gene expression profiling interactive analysis (GEPIA) bioinformatics analysis

For gene expression profiling and overall survival analysis, we conducted bioinformatics analysis on the GEPIA platform (http://gepia.cancer-pku.cn/). GEPIA is an online analysis tool for processing high-throughput RNA sequencing expression data of bulk tumorous and normal samples based on the Cancer Genome Atlas (TCGA) (https://portal.gdc.cancer.gov/) and the Genotype-Tissue Expression (GTEx, https://www.gtexportal.org/) databases. We analyzed the correlation between CXCL1 expression and the overall survival (OS) of GBM patients and the expression of CXCL1 in GBM tissues.

### sqRT-PCR

Total RNA was isolated from C6 glioma cells and its concentration and quality were determined with the help of a Nanodrop ND-1000 spectrophotometer (NanoDrop Technologies, Wilmington, DE, USA) and gel analysis. Then, the RNA samples were reverse transcribed into cDNA using a Transcriptor First Strand cDNA synthesis kit (Roche Applied Science, Penzberg, Germany). After this, semiquantitative real time PCR was performed to analyze the gene expression using the SYBR Green PCR master mix (Roche Applied Science, Mannheim, Germany). The 20 μL reaction mixture included 10 μL 2×SYBR® Premix Ex Taq (Takara, China), 0.5 μL each of 10 μM forward and reverse primers, 2 μL cDNA template and 7 μL RNase-free H_2_O. The PCR conditions included an initial denaturation step at 95°C for 5 min, followed by 45 cycles of 95°C for 10 s, 60°C for 15 s, and 72°C for 15 s. The mRNA data were normalised to GAPDH. The used primer sequences were as follows:

GAPDH **F**:CGGATTTGGCCGTATCGG **R**:TGAGGTCAATGAAGGGGTCG

CXCL1 **F**:GCAGACAGTGGCAGGGATTC **R**:CGGTTTGGGTGCAGTGGG

Cx3cl1 **F**:GAGGCAGGCAGTGGGGTTA **R**:TGGAGGCTCTGGTAGGCAAA

Serpine1 **F**:CTGCCCCGCCTCCTCA **R**:CGCCACTGTGCCGCTCT

Ddit3 **F**:CACCTCCCAAAGCCCTCG **R**:TGCTTGAGCCGCTCGTTCT

Gadd45g **F**:CCGCTGGCGTCTACGAGTC **R**:GGCTATGTCGCCCTCATCTTC

Mt1 **F**:CCAACTGCTCCTGCTCCACC **R**:GCACTTGTCCGAGGCACCTT

Rap1gap **F**:CCAGCCACAGTGGGAGCTT **R**:CCCCGGCGTCCGAATC

Klf10 **F**:AAGGAGGTTTGCTCGTTCCG **R**:GGCAGCTTCTTGGCTGATAGG

Nfkbia **F**:TGACCTGGTCTCGCTCCTGT **R**:GACGCTGGCCTCCAAACAC

### Immunofluorescence

For in situ staining, cells were washed with PBS, then fixed with 4% paraformaldehyde (PFA), washed 3× in PBS and placed in 10% normal goat serum with 0.3% Triton X-100 in PBS for 1 h. Afterwards, the cells were incubated in blocking solution with anti- NF-κB antibody overnight at 4°C. After that, the cells were washed 3× in PBS and incubated with goat anti-rabbit antibody in PBS for 1 h, washed 3× in PBS and incubated with DAPI (VIC112; VICMED, China). Finally, the cells were washed 3× with PBS and analyzed under a fluorescence microscope (IX-71; Olympus, Japan).

### Total protein, cytoplasmic and nuclear protein extraction

For total protein extraction, cells of each group were washed with ice-cold phosphate-buffered saline and lysed in RIPA lysis buffer (P0013B; Beyotime, China) containing the protease inhibitor PMSF (1Mm; VPI003; VICMED, China). Cytoplasmic and nuclear protein extraction was subsequently performed. The samples were washed with ice-cold phosphate-buffered saline and treated with a nuclear protein extraction kit (P0028; Beyotime, China) according to the manufacturer’s protocol: The cell pellet was incubated in buffer A containing PMSF and thereafter incubated for 10 min. on ice. Then, buffer B was added and the samples were incubated for another 1 min. After centrifugation at 10,000 g for 5 min at 4°C, the cytoplasmic protein (the supernatant) could be isolated. The nuclei in the pellet were isolated by further centrifugation and resuspended in nuclear extraction buffer containing PMSF for 30 min at 4°C and vortexing every 5 minutes. After centrifugation at 10,000 g for 10 min at 4°C, the supernatant contained the nuclear protein. The protein concentration was determined with the help of a BCA assay kit (P0010, Beyotime, China) and was used according to the manufacturer’s protocol. The isolated proteins were stored at −80°C until use.

### Western blot analysis

To detect the expression of specific proteins, 40 μg of total protein was separated on a SDS-polyacrylamide gel (KGP113K; KeyGEN, China) and wet transferred to a nitrocellulose transfer membrane (PALL66485F; VICMED, China). The membrane was probed with a primary and a secondary antibody. The protein bands were scanned and visualized using an Odyssey® Infrared Imaging System by LI-COR Biosciences (USA).

### Luciferase assay

To monitor the expression of the GDNF-induced *CXCL1*, we generated a luciferase expression construct containing the *CXCL1* promotor. Firstly, a Luciferase reporter gene vector was constructed by designing primers (F:CATGCTCGAGAAGAGCTGGAAGGCTTGCAGTCA; R:CATGAAGCTTGAAGTGAATCCCTGCCACTGTC) to amplify the CXCL1 promoter sequence. A Luciferase reporter gene vector (1 μg/well) and pRL-TK (50 ng/well), expressing Renilla luciferase as an internal control, were co-transfected in C6 cells (1×10^5^) and incubated for various time points. Relative luciferase activity was assessed by the Promega dual-luciferase reporter assay system (Promega, Madison, WI).

### ELISA detection

50 μl of standard, 40 μl of sample diluent and 10 μl of sample were accurately added to the wells to be tested. After gentle shaking and mixing, the plate was closed and incubated at 37°C for 30 min. Then, 50 μL developer was added into each well, gently mixed and incubated for 10 min. at 37°C and protected from light for color development. After this, the wells were washed. Finally, 50 μL termination liquid were added into each well after which eventually the absorbance of each well was measured at 450 nm [24].

### Construction of the lentiviral vector

The siRNA target was designed according to the transcripts of the r-CXCL1 gene. The siRNA sequence of the control virus vector was: TTCTCCGAACGTGCACGTAA. The interference sequence was: AGAACATCCAGTTGAAGGTGAT. The designed sequence was cloned into a pHB-U6-MCS-CMV-ZsGreen-PGK-PURO (Hanbio Biotechnology, Shanghai, China) to generate the lentiviral vector. Then, the expression vector together with the packaging vector were used to transfect 293T cells with lipofitertm (Hanbio Biotechnology, Shanghai, China). After 48 h, the supernatants containing HBLV-r-CXCL1 shRNA3-GFP-PURO and HBLV-GFP-PURO NC lentiviruses were collected. After that, the lentiviruses were purified by ultracentrifugation after which the virus titers were detected.

### ChIP assay

A ChIP Chromatin Immunoprecipitation kit (Millipore, Bedford, MA, USA) was employed for the ChIP assay. Briefly, C6 cells were fixed with 1% formaldehyde for 10 min at 25°C and then washed with ice-cold phosphate-buffered saline buffer containing protease inhibitors. The nuclei were collected and resuspended in SDS lysis buffer containing protease inhibitor after which they were sonicated to generate crosslinked DNA fragments with 200–1000 bp length. The soluble supernatant was obtained after brief centrifugation whereupon it was incubated with Protein G Agarose. An aliquot of the supernatant was saved as a DNA input control and the remainder was incubated with anti-NF-κB antibody (Cell Signaling Technology, USA). A rabbit IgG antibody was used as a negative control. Immunoprecipitation was carried out overnight at 4°C and immune complexes were collected using Protein G Agarose beads. The immune complexes and the input were eluted and protein-DNA complexes were de-crosslinked overnight at 65°C. ChIP DNA was purified and subjected to PCR, which amplified the *CXCL1* promoter sequence containing putative SRY-related protein-binding sites (site 1: position +951bp to +1116bp; site 2: position +356bp to +511bp; site 3: position +771 bp to +940bp). Specific ChIP primers used for PCR were as follows: CXCL1-1; F: TTGGGAGTGGAGCAAGGGG; R: TGGAGCTGGTTTAGGATCTGAGTC; CXCL1-2; F: CGTCCTCAGCCCAGAAAAAAC; R: CTCTTCTTTGCTTTTTGAACTCGG; CXCL1-3; F: TGGAGTCCTAGGTGGCGTGG; R: TTTTTGCTTTTTGCCCCAAAGTC.

### Cell viability assessment

For cell viability assays, 1×10^3^ cells were plated in clear bottom 96-well plates, processed according to the manufacturer’s instructions using a Cell Counting Kit-8 (CCK-8; CK04; Dojindo, Japan) and afterwards quantified on a microplate reader (Synergy2; BioTek, USA).

### Assessment of cell viability

C6 cells (1×10^3^ cells/well) were cultured for 12 h in 96-well plates before being treated with different concentrations of rapamycin BAY 11–7082 for 24 h. Cell viability was evaluated using an 3-(4,5-dimethylthiazol-2-yl)-2,5-diphenyltetrazolium bromide (MTT) assay with absorbance measurement at 490 nm.

### Statistical analysis

Statistical analysis was performed using SPSS 19.0 for Windows. The data are presented as the mean ± standard deviation obtained from three independent experiments. One-way ANOVA and Tukey’s post-hoc analysis were used to determine the differences between groups. *P* < 0.05 was considered to be statistically significantly different.

## Results

### 1 Bioinformatics-based screening of differentially expressed genes (DEGs) related to GDNF-induced proliferation of C6 cells

**Fig 1:**
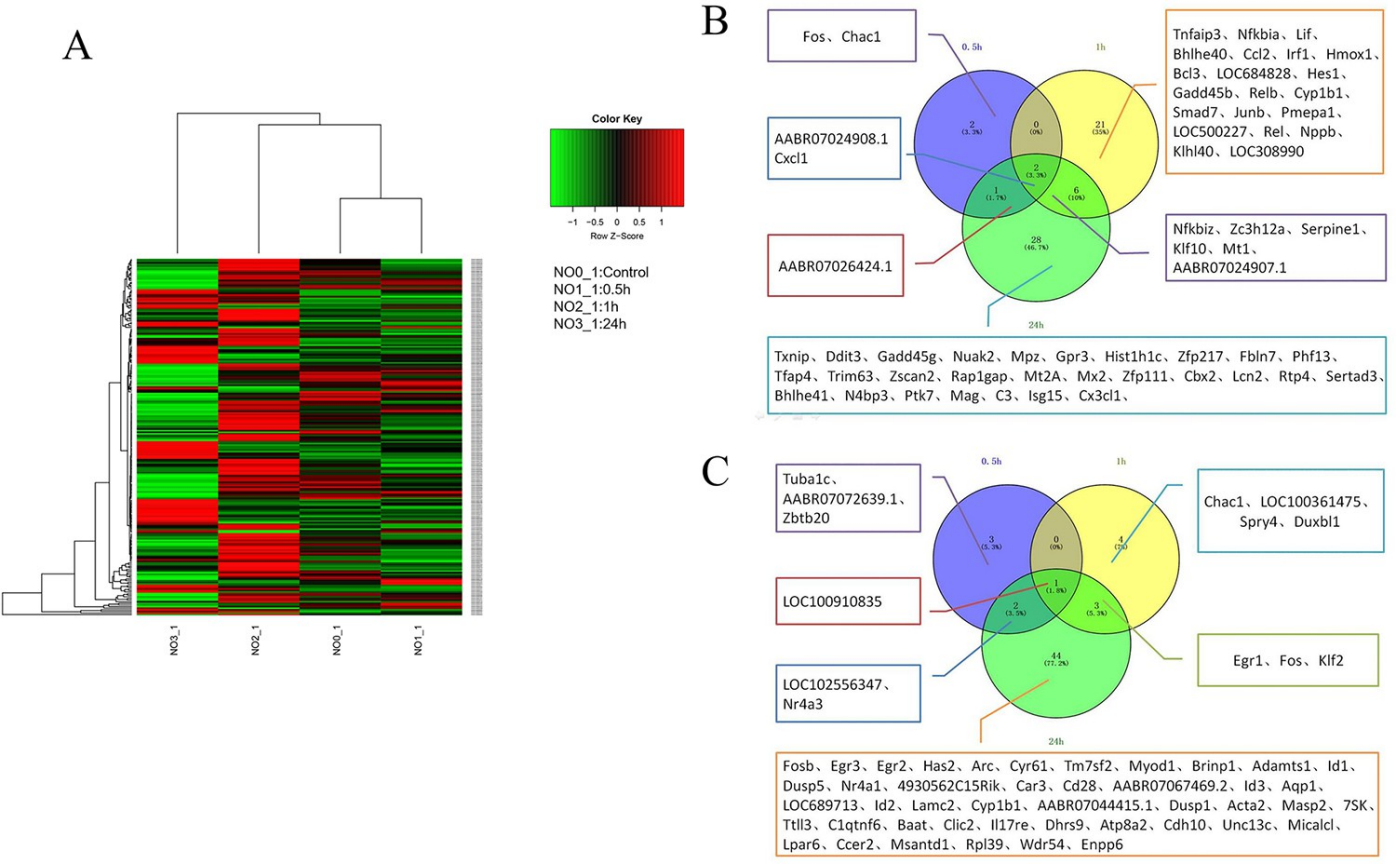
The distribution of DEGs were showed by heat map and Venn diagram. The heat map of DEGs. The abscissa indicated sample numbers, and NO0_1, NO1_1, NO2_1, NO3_1 represented the total RNA samples extracted from C6 glioma cells after exogenous GDNF treated 0, 0.5, 1 and 24 hours, respectively. The ordinate represented the gene name. The color key in the upper right corner indicated the color range, in which red indicated the up-regulated DEGs, and green indicated the down regulated DEGs. (B) Three sets of Venn plots showed the DEGs were up-regulated in C6 cells at 0.5h, 1 and 24h after GDNF treatment compared with 0 h. (C) Three sets of Venn plots showed the DEGs were down-regulated in C6 cells at 0.5h, 1 and 24h after GDNF treatment compared with 0 h.

It has shown that GDNF can significantly increase the proliferation and migration of glioma cells [15]. In order to elucidate the molecular mechanism and target underlying the GDNF-induced C6 glioma cell proliferation, the C6 cells were incubated with 40 ng/ml GDNF [15] for 0 h, 0.5 h, 1 h, and 24 h, respectively. The total RNA of the samples was extracted and sequenced by RNA-Seq. The results showed that the numerical regions of the four groups of samples were of high quality, with balanced distribution of bases (**S1A and S1B Fig**). The percentage of tested genes increased with increasing sequencing depth, and the correlation between samples was high (**S1C and S1D Fig**). Differentially expressed genes are displayed by a heat map drawn with the help of the R language software package. Compared to the 0 h group, 11, 37, and 87 genes were differentially expressed (DEG) in the 0.5 h, 1 h, and 24 h group, respectively, of which 5, 29, and 37 were up-regulated and 6, 8, and 50 were down-regulated (**Fig 1A**). In order to find the intersection of DEGs, we used Venny’s online software to draw a Venn diagram. There were two common up-regulated DEGs (CXCL1 and AABR07024908.1) and one common down-regulated DEG (LOC100910835) in the 0.5, 1, and 24 h group (**Fig 1B and 1C**).

**Fig 2:**
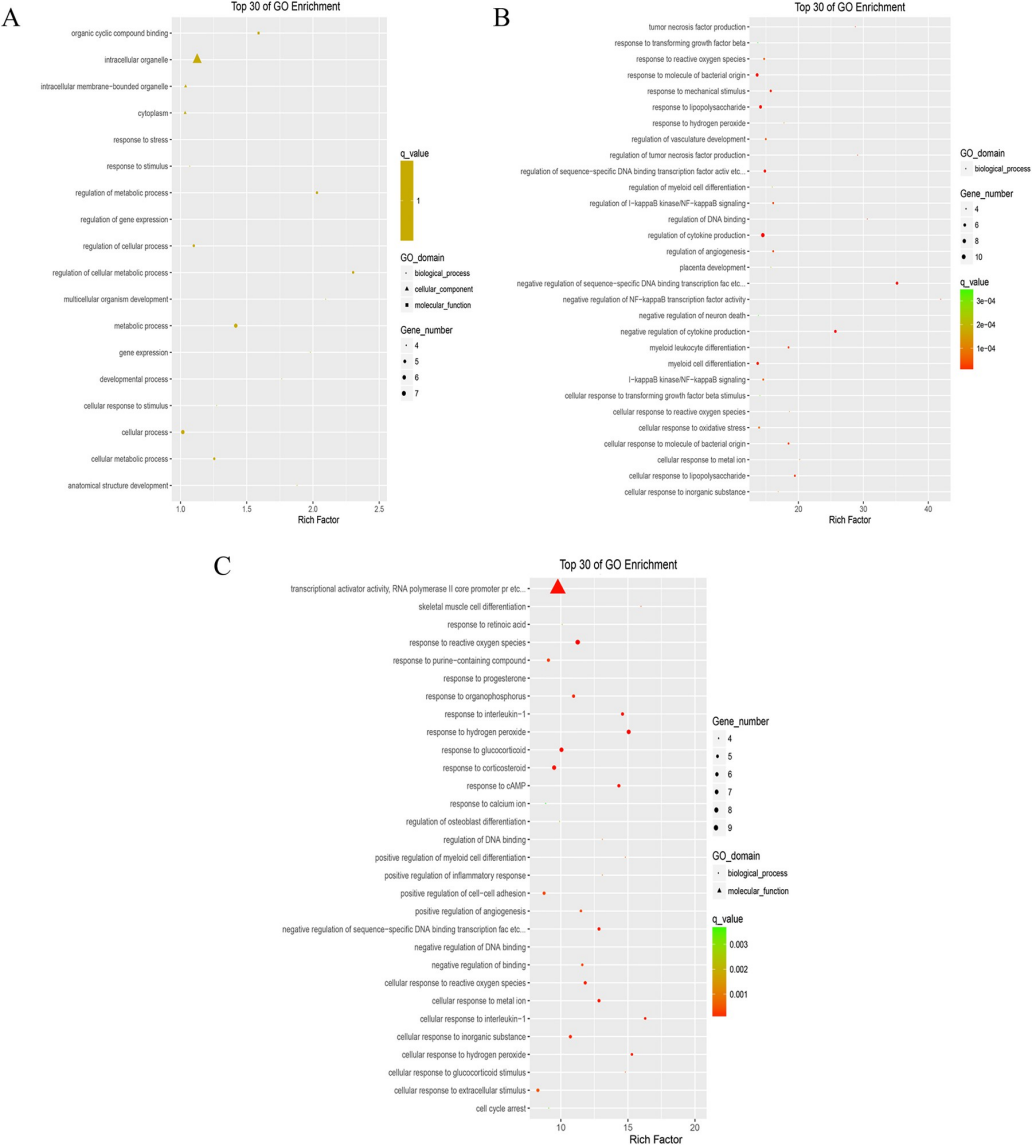
Go function enrichment scatter of DEGs. DEGs were enriched for GO function and the scatter diagram of the top 30 was drawn after exogenous GDNF treated 0.5 (A), 1 (B) and 24 h (C) compared with 0 h respectively.

**Fig 3:**
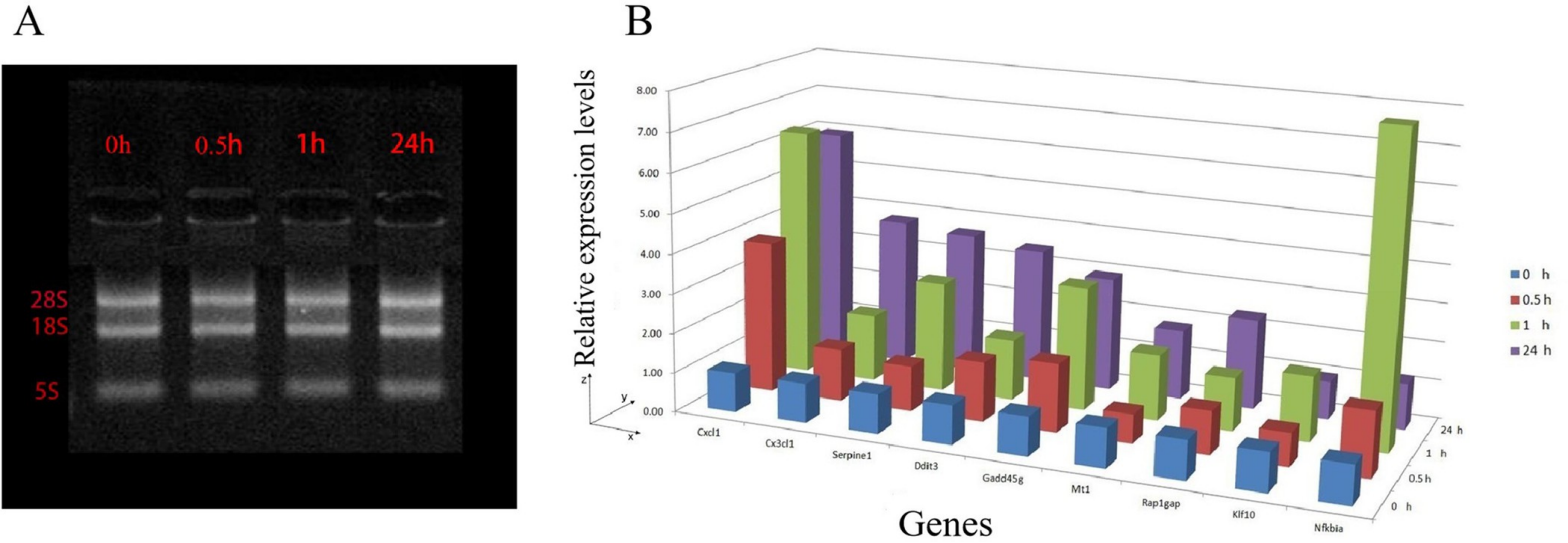
The relative expression of genes was detected by sqPCR. (A) RNA integrity was detected by gel electrophoresis. (B) The relative expression of genes was detected by sqPCR.

Furthermore, the DEGs were enriched for GO function and KEGG analysis. The number of GO terms mapped resulted in 18, 325, and 461 in the 0.5, 1, and 24 h group, respectively (**Fig 2**). Then, the David software was used to enrich DEGs with KEGG. The results showed that 2 of the 5 DEGs were found to be located on the KEGG pathway. However, the enriched KEGG pathway resulted in 0 in the 0.5 h group (thresholds: count = 2, ease = 0.1) (**S2 Fig**); However, 20 of the 29 DEGs were found to be located on the KEGG pathway and the enriched KEGG pathway resulted in 7 in the 1 h group (thresholds: count = 2, ease = 0.1) (**S3 Fig**); 16 of the 37 DEGs genes were found to be located on the KEGG pathway with the enriched KEGG pathway result of 1 in the 24 h group (thresholds: count = 2, ease = 0.1) (**S4 Fig**). Finally, STRING and Cytoscape software was used to analyze the protein-protein interaction (PPI) network of DEGs and further module analysis. The results showed that rich protein interactions only existed in the 1 h group and that there were mainly four functional modules: the TNF signaling pathway, the NF kappa B signaling pathway, the I-kappa B/NF-kappa B complex, and the beta interferon production pathway (**S5 Fig**). In order to validate the RNA-seq results, gel electrophoresis was performed to prove the integrity of RNA at this time. **Fig 3A** showed that the 18S and 28S bands of the target RNA were clear under agarose gel electrophoresis, indicating that the RNA is relatively complete and not degraded. In addition, for some genes a sqPCR analysis was carried out. As a result, the relative expression level of CXCL1 could be shown to be significantly increased in the 0.5, 1, and 24 h group (**Fig 3B**). Because CXCL1 was up-regulated in the DEG analysis and is known to be involved in cellular proliferation in GO gene annotation (GO; 0008283), CXCL1 turned out to be the most noteworthy target gene.

### 2 CXCL1 played a pivotal role in the proliferation of C6 cells and in GBM

**Fig 4:**
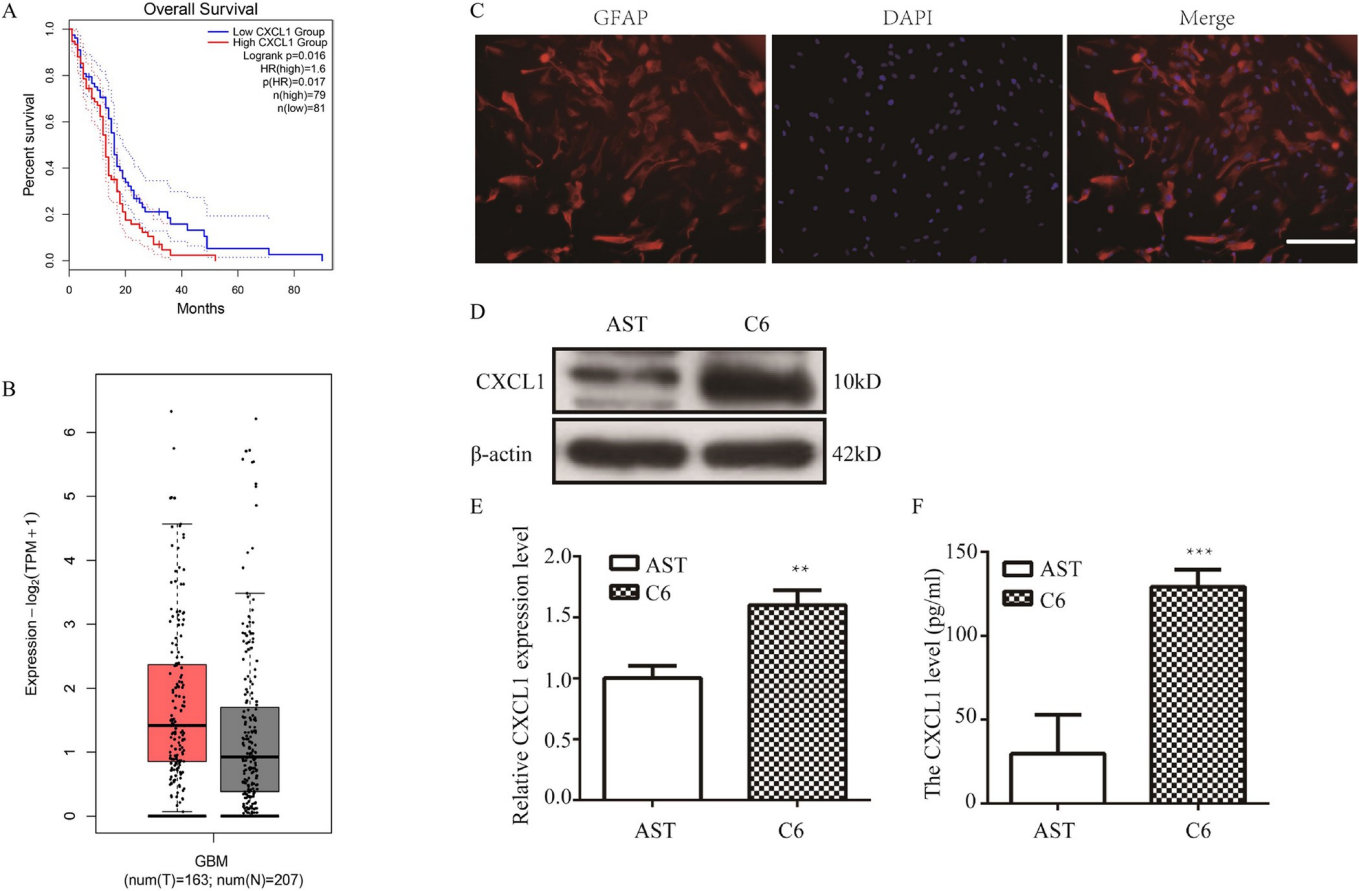
The expression level of CXCL1 in C6 cells and GBM. (A) The correlation between CXCL1 and the overall survival of GBM patients on GEPIA. (B) The expression of CXCL1 in GBM tissues compared with related normal tissues. (C) Identification of AST. AST transmitted to the third generation were stained with anti-GFAP immunofluorescence. The cytoplasm of positive cells was stained red, and the nucleus of DAPI stained blue. Bar = 200 μm. (D) Western blot showed that CXCL1 protein expression level in C6 cells and AST. (E) The statistical results of (D). (F) ELISA showed that CXCL1 supernatant content in C6 cells and AST. (**P<0.01, ***P<0.001).

We used RNA-seq combined with bioinformatics technology to screen CXCL1 as a GDNF-induced proliferation-related gene which could not be confirmed by the experiments. Present studies show that CXCL1 acts as a carcinogen in glioma which promotes the proliferation and migration of glioma cells [19]. Therefore, with the help of the GEPIA platform (http://gepia2.cancer-pku.cn/#survival), we firstly analyzed the correlation between CXCL1 expression and the overall survival of GBM patients. We found that CXCL1 was negatively correlated to the OS of GBM patients (**Fig 4A**). In addition, we found that the expression of CXCL1 was up-regulated in GBM tissues compared to normal tissues (**Fig 4B**). To explore the expression and secretion of CXCL1, protein and supernatant from AST and C6 cells were extracted. AST cells were obtained by isolation and purification of rat primary cells with a positive GFAP rate in the cytoplasm of higher than 95% (**Fig 4C**). Western blot and ELISA results showed that the CXCL1 protein expression level and the supernatant content in C6 cells were significantly higher than those in rat primary astrocytes, respectively (**Fig 4D–4F**). These results demonstrated that CXCL1 was up-regulated in C6 cells and GBM.

**Fig 5:**
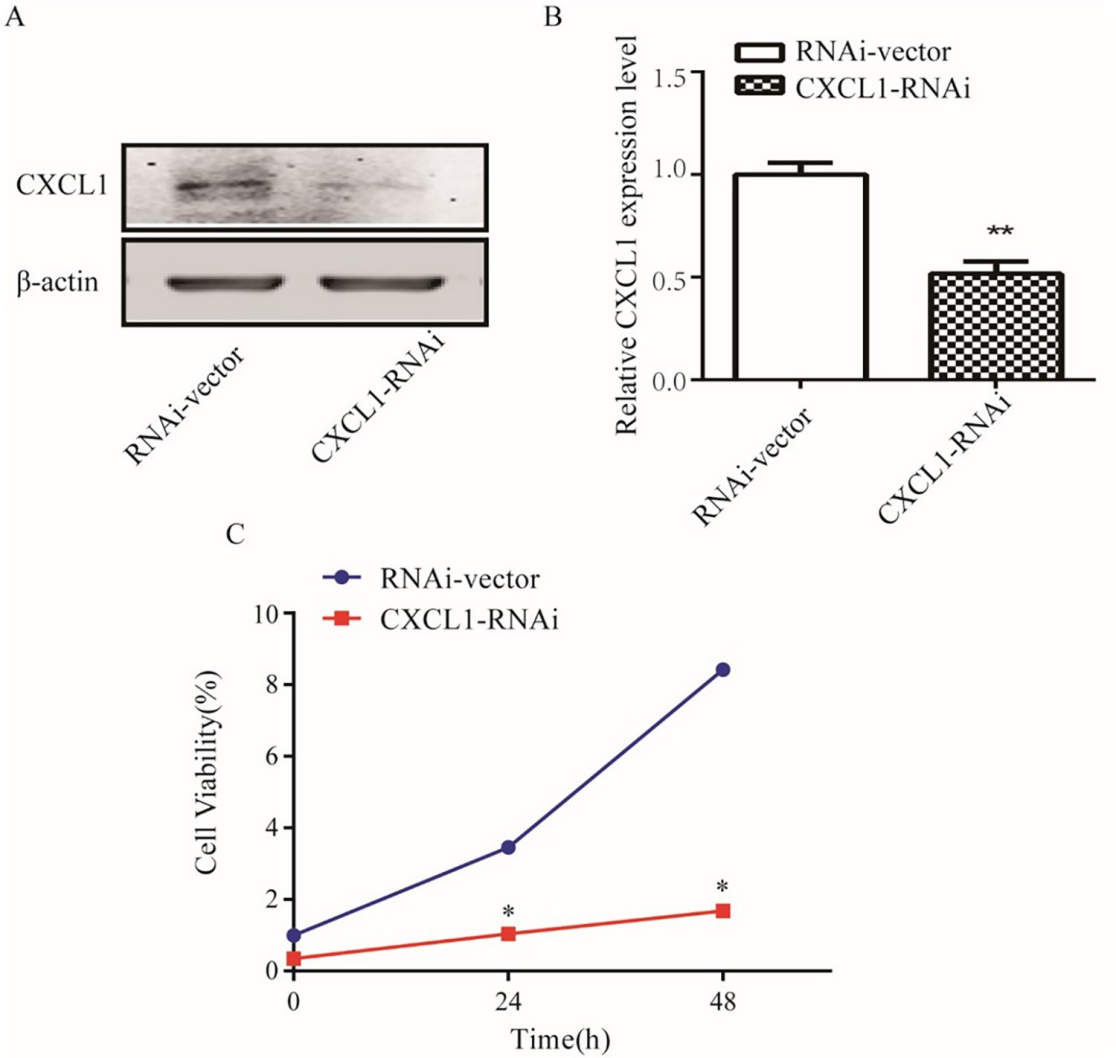
The effect of CXCL1 on proliferation of C6 cells. (A) Western blot showed that the expression level of CXCL1 in the CXCL1 knockdown group (CXCL1-RNAi) and the control group (RNAi-vector). (B) The statistical results of (A). (C) CCK-8 showed that the OD450 value in CXCL1-RNAi and RNAi-vector group. (*P<0.05, **P<0.01).

To further validate the effect of CXCL1 on the proliferation of C6 glioma cells, we constructed a lentiviral vector with an interfering RNA (RNAi) targeting the rat CXCL1 gene and which was used to infect C6 cells. Western blot results showed that the expression level of CXCL1 was significantly down-regulated in the CXCL1 knockdown group (CXCL1-RNAi) compared to the control group (RNAi-vector) (**Fig 5A and 5B**). The CCK-8 assay results showed that the OD450 value of the CXCL1-RNAi group was lower than that of RNAi-vector group (**Fig 5C**). The above results suggest that knockdown of CXCL1 inhibits the proliferation of C6 cells.

### 3 CXCL1 mediated the proliferation of C6 cells induced by exogenous GDNF

**Fig 6:**
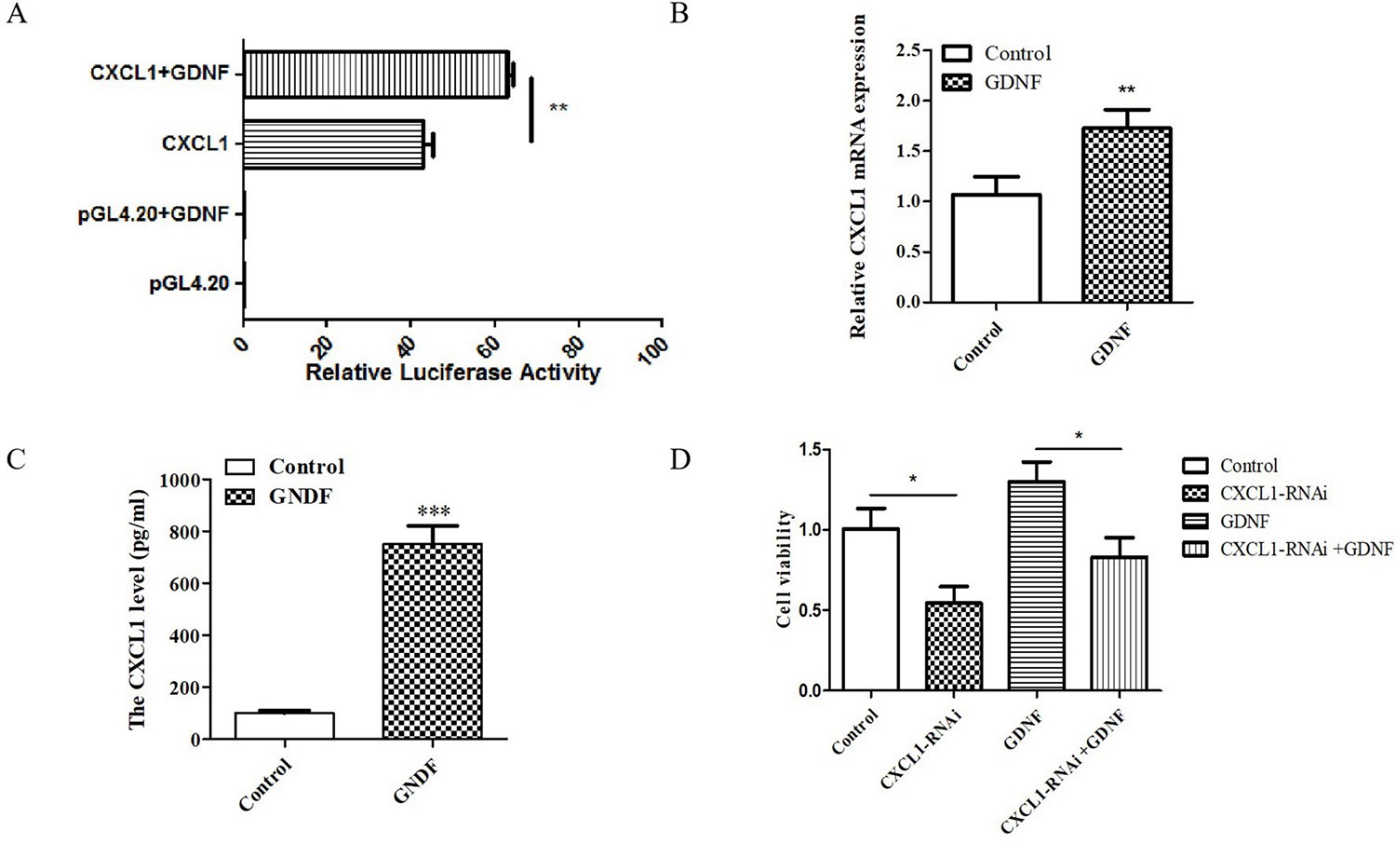
CXCL1 mediated the proliferation of C6 cells induced by exogenous GDNF. (A) CXCL1 promoter activity increased significantly after GDNF treatment by double luciferase reporter gene activity. pGL 4.20 was an empty carrier. pRL-TK was internal reference. (B) The mRNA level of CXCL1 was analyzed by q-PCR. (C) Cell supernatants were collected and the secretion level of CXCL1 was detected by ELISA. (D) CCK-8 was used to detect the cell activity of each group. (*P<0.05, **P<0.01,***P<0.001).

In order to detect the effect of exogenous GDNF on CXCL1, we used a double luciferase reporter gene activity assay to detect the effects of GDNF on the CXCL1 promoter region. The results showed that compared to the CXCL1 group, the CXCL1 promoter activity increased significantly after GDNF treatment (**Fig 6A**).

To validate the effect of GDNF on CXCL1, exogenous GDNF (40 ng/mL) was added to C6 cells and the CXCL1 mRNA level was analyzed by sqPCR. In addition, cell supernatants were collected and the secretion level of CXCL1 was detected by ELISA. The sqPCR results showed that GDNF could promote the expression of CXCL1 and ELISA results showed that GDNF could significantly promote the secretion of CXCL1 in C6 cells (**Fig 6B and 6C**).

In order to prove that CXCL1 is able to mediate the proliferation of C6 cells after GDNF-induction, C6 cells were infected with a CXCL1 knock-out lentiviral vector. After administration of exogenous GDNF (40 ng/mL) to the C6 cells, CCK-8 was used to detect the cell activity of each group. Compared to the control group, the activity of the C6 cells in the CXCL1-RNAi group was decreased. Moreover, compared to the GDNF treatment group, the C6 cell viability was decreased in the CXCL1-RNAi and the exogenous GDNF co-treatment group (**Fig 6D**). These results demonstrate that CXCL1 mediates the proliferation of C6 cells after exogenous GDNF administration.

### 4 GDNF promoted NF-κB nuclear translocation and the interaction of CXCL1 with NF-κB

**Fig 7:**
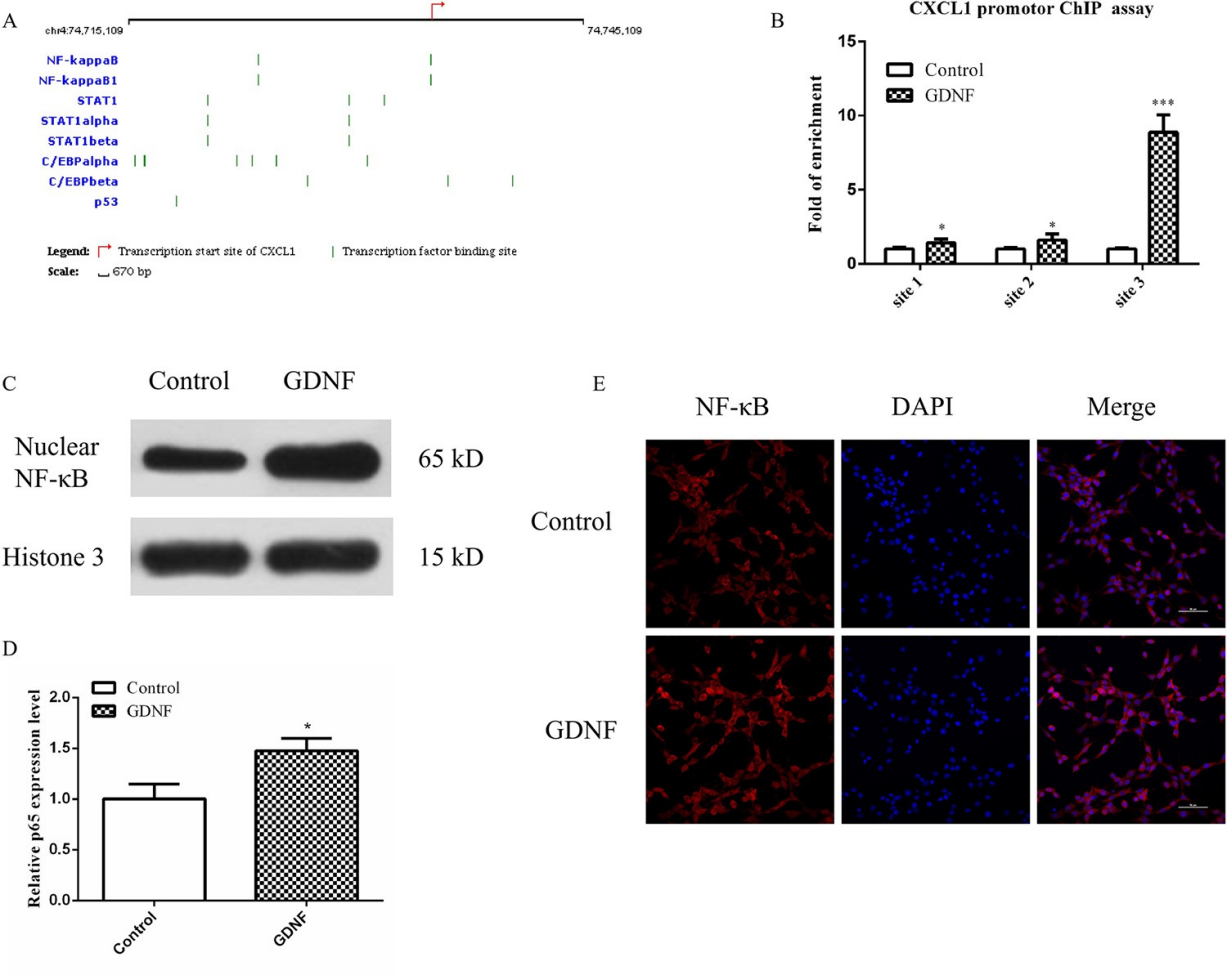
GDNF promoted NF-κB nuclear transformation and the interaction CXCEL with NF-κB. (A) Transcription factors that regulate the gene CXCL1. Qiagen online software was used to analyze the TF that initiated CXCL1 transcription and its corresponding BS. (B) Exogenous GDNF promoted the interaction CXCEL with NF-κB by ChIP-qPCR assay. (C) Western blot showed exogenous GDNF promoted NF-κB nuclear transformation. The C6 cells were treated with exogenous GDNF for 48h, and nuclear protein were extracted to determine NF-κB (p65) levels by Western blot. Histone 3 was used as internal references. (D) The statistical results of (C). (E) NF-κB nuclear transformation was analyzed by Immunofluorescence. The C6 cells were treated with exogenous GDNF for 48h and fixed with 4% paraformaldehyde (PFA) for Immunofluorescence. Bar = 200 μm. (*P<0.05,**P<0.01, ***P<0.001).

In order to explore the possible mechanism of the GDNF promoted C6 cell proliferation via promotion of the CXCL1 gene transcription, we used a Qiagen online software to analyze the transcription factor (TF) that initiates the CXCL1 transcription at its corresponding binding sites (BS). The results showed that the most relevant transcription factors were NF-κB, NF-κB1, STAT1, STAT1 α, STAT1 β, C/EBP α, C/EBP β and P53, in which the TF-BS positions of NF kappa B on the CXCL1 gene promoter were NC_74723660, NC_74735035, and NC_74735036, respectively (**Fig 7A**). In most tumors, NF-κB is abnormally activated and regulates various properties of cancer cells, including proliferation, invasion, inhibition of apoptosis, and angiogenesis [25]. Accordingly, we designed and synthesized three primers able to bind to the CXCL1 promoter region and used the ChIP-qPCR assay to study the binding sites of transcription factor NF-κB in the CXCL1 promoter region in the control and GDNF treatment group. The results indicated that site 1 (promoter I + 951 BP to + 1116 bp), site 2 (promoter I + 356 BP to + 511 bp), and site 3 (promoter I + 771 BP to + 940 bp) were significantly correlated to the CXCL1 promoter regions after GDNF treatment with the highest binding affinity to site 3 (**Fig 7B**).

To clarify the effect of GDNF on nuclear translocation of NF-κB in C6 cells, we used a Western blot analysis. The results showed that the expression level of NF-κB in the GDNF group was higher than that in the control group. Additionally, we performed immunofluorescence stainings and found that exogenous GDNF promoted the translocation of NF-κB to the nucleus (**Fig 7C–7E**). These results suggest that GDNF promotes NF-κB nuclear translocation and identified an interaction of CXCL1 with NF-κB.

### 5 NF-κB mediated the effect of GDNF on CXCL1-induced proliferation in rat C6 glioma cells

**Fig 8:**
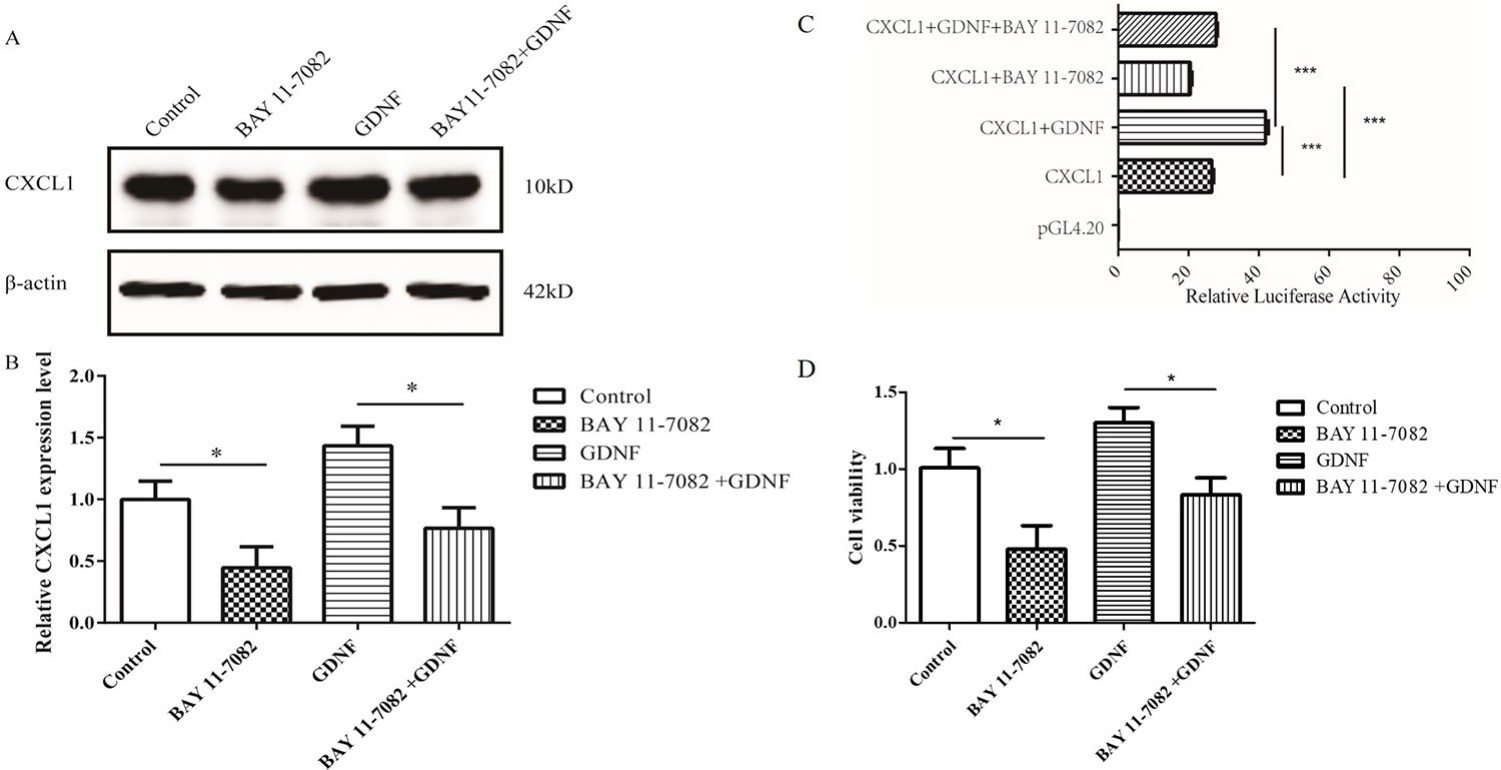
NF-κB mediated the effect of GDNF on CXCL1 and proliferation in rat C6 glioma cells. (A) Western blot showed that NF-κB mediated the effect of exogenous GDNF on CXCL1. (B) The statistical results of (A). (C) NF-κB mediated GDNF enhanced the activity of CXCL1 gene promoter region by double luciferase reporter gene activity. pGL 4.20 was an empty carrier. pRL-TK was internal reference. (D) CCK-8 results showed NF-κB mediated the proliferation of C6 cells by exogenous GDNF. (*P<0.05, **P<0.01, ***P<0.001).

In order to further study the role of NF-κB in the proliferation of C6 cells induced by GDNF, we used BAY 11–7082, a NF-κB specific inhibitor that decreases the phosphorylation level of IκB-α. Here, it was observed that C6 cells may tolerate up to 10 μM of BAY 11–7082 in the MTT assay, and the expression of NF-κB in nucleus was suppressed in the BAY 11-7082-treated cells (**S6 Fig**). Western blot results showed that the expression level of CXCL1 protein in the BAY 11–7082 treated group was significantly lower than that of the control group. Compared to the GDNF group, the expression level of CXCL1 protein was decreased in the BAY 11–7082 and the exogenous GDNF co-treatment group (**Fig 8A and 8B**). These results suggest that NF-κB is able to mediate the effect of exogenous GDNF on CXCL1.

Previous experimental results showed that exogenous administration of GDNF enhanced the activity of the CXCL1 promoter. In order to further clarify the role of NF-κB in this process, we used a double luciferase reporter gene assay. The results indicated that compared to the CXCL1 group, the CXCL1 promoter activity was significantly decreased after treatment with BAY 11–7082, suggesting that BAY 11–7082 is able to inhibit CXCL1 promoter activity. Compared to the GDNF group, the CXCL1 promoter activity was also significantly down-regulated in the BAY 11–7082 and the exogenous GDNF co-treatment group (**Fig 8C**). These results demonstrate that the GDNF enhanced CXCL1 promotor activity can be inhibited by BAY 11–7082.

Furthermore, we investigated the effect of GDNF on the proliferation in rat C6 glioma cells. CCK-8 results showed that the proliferation ability of C6 cells in the BAY 11–7082 group was lower than that of the control group. Compared to the GDNF group, the proliferation ability of C6 cells in the BAY 11–7082 and GDNF co-treatment group was inhibited (**Fig 8D**). These results indicate that NF-κB mediates the exogenous GDNF-induced proliferation of C6 cells.

## Discussion

Glioblastomas are histologically and genetically heterogeneous tumors whose mechanism of tumorigenesis is uncertain [26, 27]. Studies have shown that an important link in the pathogenesis of GBM is the uncontrolled proliferation of glioma cells [28]. By exploring growth factors and intracellular signaling pathways associated with glioma cell proliferation, we were able to identify clinical targets that block the proliferation of GBM, thus providing a theoretical basis for the diagnosis and treatment of GBM.

Recent studies have shown that GDNF is associated with the development of gliomas and other cancers [29–32]. In 1994, Lin et al. isolated GDNF from cells of the rat B49 glioma line for the first time [33]. Since then, it has also been reported that GDNF is highly expressed in malignant glioma tissues and cell lines such as C6 [12, 34], U87 [35] and U251 [36, 37]. Compared to normal brain tissues, GDNF levels in glioma tissues are increased by five times [12]. Studies have shown that GDNF significantly induces glioma cell proliferation and migration [11, 13, 14], while knocking down the expression of GDNF or its receptor GDNF family receptor alpha 1 (GFR alpha 1) could effectively inhibit the pathological progress of glioma [38]. In the previous experiments, our team confirmed that exogenous GDNF is able to promote the proliferation of rat glioma C6 cells in vitro [15].

However, little research has been carried out with regard to the mechanism of high secretory GDNF in GBM glioma tumorigenesis. Preliminary data of our group suggest that Neuropilin-1 (NRP1) may be a membrane receptor that mediates the proliferation of C6 glioma cells after exogenous GDNF administration [15]. This process seems to be mediated by up-regulating cyclins PCNA and Ki-67 [39], however, obviously, these were not sufficient.

In order to clarify the GDNF gene target responsible for promoting C6 glioma cell proliferation, we employed RNA-Seq analysis. Comparing the distribution of DEGs in the 0.5, 1, and 24 h group with the 0 h group, the results suggested two common up-regulated DEGs: AABR07024908.1 and CXCL1. Since AABR07024908.1 has not been annotated in the existing gene annotation system and CXCL1 was related to cell proliferation (GO: 0008283) in GO gene annotation, we hypothesized that CXCL1 may be the most noteworthy target gene.

Then, we found that the expression of CXCL1 was up-regulated in GBM tissues compared to related normal tissues and that CXCL1 was negatively correlated to the OS of GBM patients. Western blot and ELISA results showed that the CXCL1 protein expression level and supernatant content of C6 cells were significantly higher than those of rat primary astrocytes, respectively (**Fig 4**). To further verify the effect of CXCL1 on the proliferation of C6 glioma cells, we constructed an interfering RNA (RNAi) lentiviral vector targeting the rat CXCL1 gene. The CCK-8 kit experiments showed that knockdown of CXCL1 inhibited the proliferation of C6 cells (**Fig 5)**.

Studies have shown that the stimulation of GDNF-RET in immune cells was able to cause the up-regulation of the chemokine CXCL1 [40]. In order to detect the effect of exogenous GDNF on CXCL1 levels, we used a double luciferase reporter gene activity assay to detect the effects of GDNF on the CXCL1 promoter. The results showed that the CXCL1 promoter activity increased significantly after GDNF treatment (**Fig 6A**). In addition, by sqPCR and ELISA we demonstrated that exogenous GDNF was able to increase the expression and secretion of CXCL1 in C6 cells (**Fig 6B and 6C**). Compared to the GDNF-treated group, the viability of C6 cells was decreased in the co-treatment group of exogenous GDNF and CXCL1-RNAi (**Fig 6D**). Therefore, CXCL1 indeed mediated the effect of exogenous GDNF on C6 cell proliferation.

How does GDNF mediate the transcriptional regulation of CXCL1? By using the Qiagen online software tool, we tried to shed some light on this by selecting the transcription factor NF-κB as a target. We could show that NF-κB existed in almost all animal cell types and was involved in the response of cells to stimuli such as stress, cytokines, free radicals, heavy metals, ultraviolet radiation, oxidative LDL, and bacterial or viral antigens [41]. It has been documented that activation of NF-κB is an important driver of a malignant phenotype in GBM patients [42]. NF-κB p65/p52 mediated GDNF expression and stem cell migration [43, 44]. Accordingly, we used the ChIP-qPCR assay to prove that NF-κB was significantly correlated to binding to CXCL1 promoter region site 3 after GDNF treatment (**Fig 7B**) and that exogenous GDNF promoted the translocation of NF-κB into the nucleus (**Fig 7C–7E**).

To further study the role and mechanism of NF-κB in GDNF-induced CXCL1-mediated C6 cell proliferation, we used BAY 11–7082, a phosphorylation inhibitor of NF-κB. Compared to the GDNF treated group, the expression level of CXCL1 protein was lower than that in the BAY 11–7082 and the GDNF co-treated group (**Fig 8A and 8B**). Next, we studied the role of NF-κB in the proliferation of C6 cells promoted by GDNF. The results showed that the enhancement of CXCL1 promoter activity by GDNF could be inhibited by BAY 11–7082 (**Fig 8C**). Finally, CCK-8 experiments revealed that compared to the GDNF treatment group, the proliferation of C6 cells was reduced in the BAY 11–7082 and GDNF co-treatment group (**Fig 8D**). It is suggested that NF-κB mediates the proliferation of C6 cells after exogenous GDNF administration. Thus, we hypothesize that NF-κB mediates the proliferation of C6 cells by exogenous GDNF administration via interaction with the promoter region of the CXCL1 gene, thereby promoting its transcriptional activity.

In this study, we explored the relationship between GDNF, NF-κB, CXCL1, and cell proliferation and proved that GDNF promoted C6 glioma cell proliferation through the NF-κB/CXCL1 signaling pathway. Our work not only enriched the research on the mechanism of glioma tumorigenesis but also provides a new idea for a targeted therapy of malignant glioma.

## Supporting information

S1 Fig Evaluated the quality of sequenced samples. (A) The sequenced Q-value box diagram. The X-axis was the base position in reads, and the Y-axis represented the mass value of all bases. The green, yellow and red backgrounds represented the high, reasonable and low quality of the numerical area respectively. The blue line represented the average value of the quality value. (B) Base distribution map of sequencing. The X-axis was the position of each base in reads, and the Y-axis was the proportion of each base. Red: T; Blue: C; Green: A; Black: G. (C) Saturation analysis diagram of four samples groups. X-axis was the sequencing depth (unit: thousands of reads); The Y-axis was the percentage of genes covered. NO0_1: GDNF 0 h group; No1_1: GDNF 0.5 h group; No2_1: GDNF 1 h group; No3_1: GDNF 24 h group. (D) Heat map of gene expression correlation of four samples groups. The color depth represented the correlation, and the value was the correlation coefficient.
(TIF)

S2 Fig KEGG function note table for DEGs in the group of exogenous GDNF for 0.5 h. (TIF)

S3 Fig KEGG function note table for DEGs in the group of exogenous GDNF for 1 h. (A) KEGG function note table. (B) DEGs enriched KEGG table.
(TIF)

S4 Fig KEGG function note table for DEGs in the group of exogenous GDNF for 24 h. (A) KEGG function note table. (B) DEGs enriched KEGG table.
(TIF)

S5 Fig Analysis of protein-protein interaction of DEGs by STRING protein interaction network. (A) The group of exogenous GDNF for 0.5 h. (B) The group of exogenous GDNF for 1 h. (C) The group of exogenous GDNF for 24 h.
(TIF)

S6 Fig The expression level of nuclear NF-κB in BAY 11–7082 treated C6 cell. (A) Cytotoxicity of BAY 11–7082 to C6 cells. (B) Western blot showed that the expression level of CXCL1 in the BAY 11–7082 treated group and the control group. (C) The statistical results of (A). (*P<0.05, ***P<0.001).
(TIF)

S1 Raw images (PDF)
