# Supplementary figures and images for: GDNF triggers proliferation of rat C6 glioma cells via the NF-κB/CXCL1 signaling pathway
Source: PLoS One. 2023 Aug 18;18(8):e0289071. doi: 10.1371/journal.pone.0289071 (PMC10437914; doi:10.1371/journal.pone.0289071)

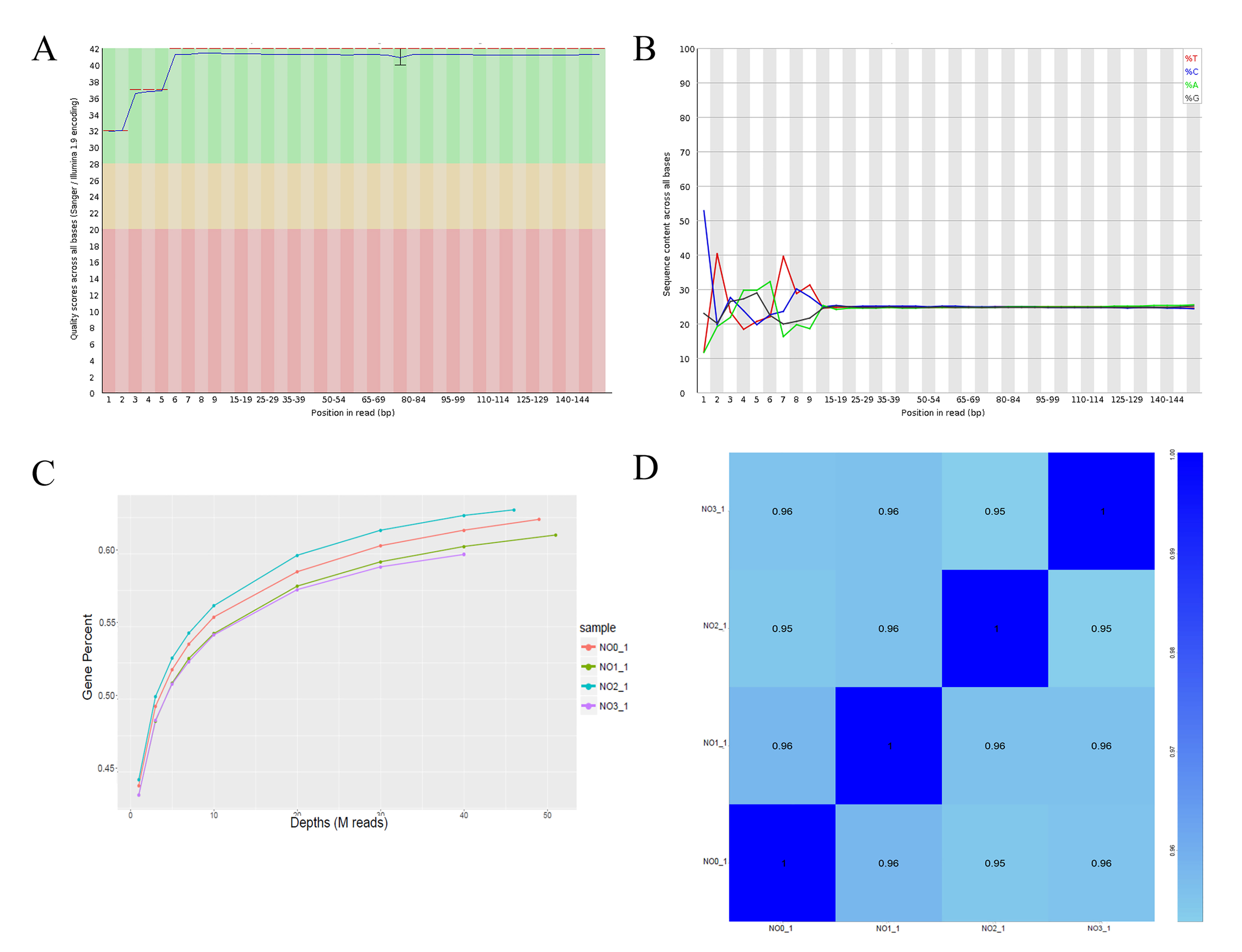

Supplement: S1 Fig — (A) The sequenced Q-value box diagram. The X-axis was the base position in reads, and the Y-axis represented the mass value of all bases. The green, yellow and red backgrounds represented the high, reasonable and low quality of the numerical area respectively. The blue line represented the average value of the quality value. (B) Base distribution map of sequencing. The X-axis was the position of each base in reads, and the Y-axis was the proportion of each base. Red: T; Blue: C; Green: A; Black: G. (C) Saturation analysis diagram of four samples groups. X-axis was the sequencing depth (unit: thousands of reads); The Y-axis was the percentage of genes covered. NO0_1: GDNF 0 h group; No1_1: GDNF 0.5 h group; No2_1: GDNF 1 h group; No3_1: GDNF 24 h group. (D) Heat map of gene expression correlation of four samples groups. The color depth represented the correlation, and the value was the correlation coefficient. (TIF) [file pone.0289071.s001.tif]

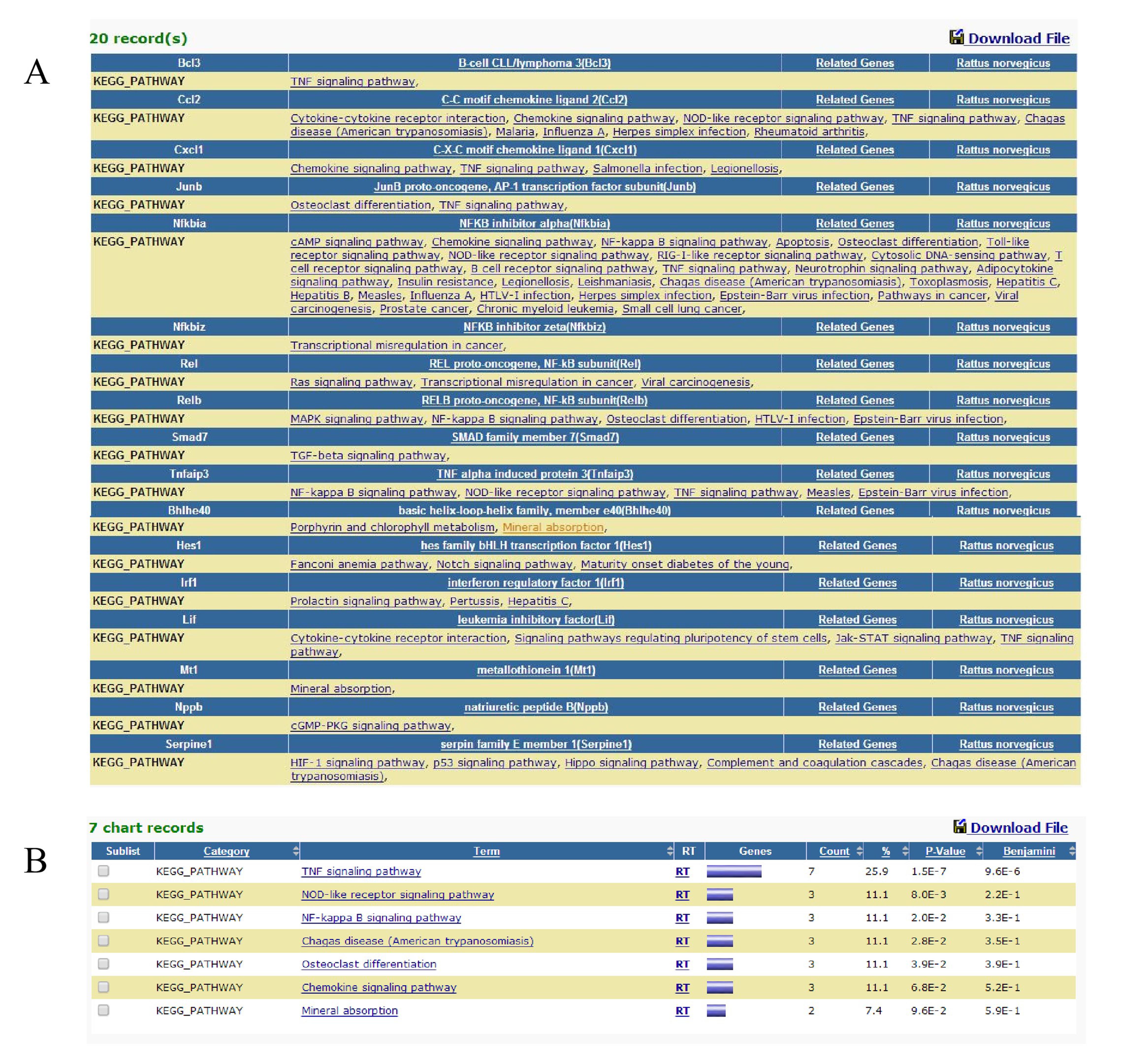

Supplement: S2 Fig — (TIF) [file pone.0289071.s002.tif]

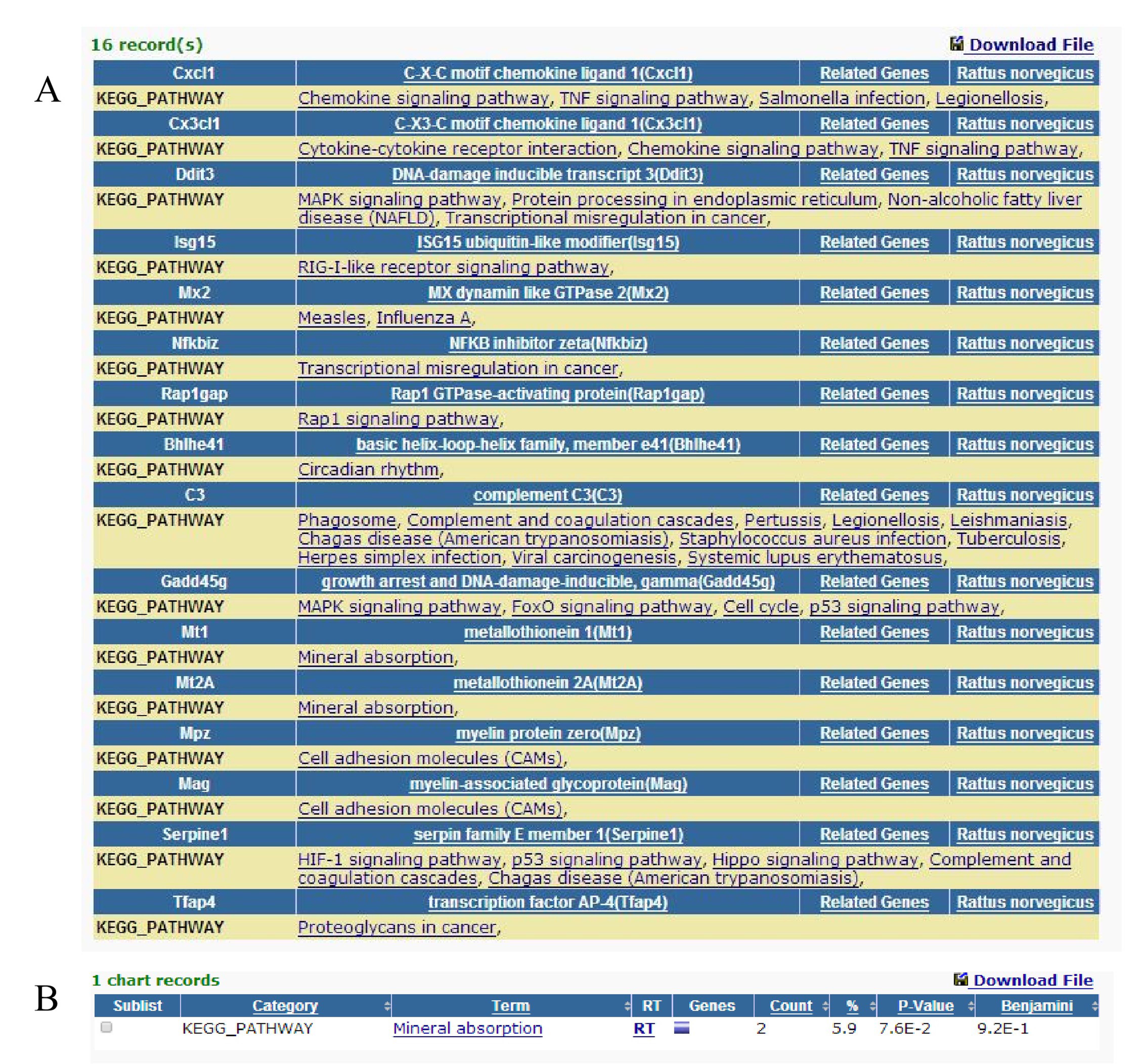

Supplement: S3 Fig — (A) KEGG function note table. (B) DEGs enriched KEGG table. (TIF) [file pone.0289071.s003.tif]

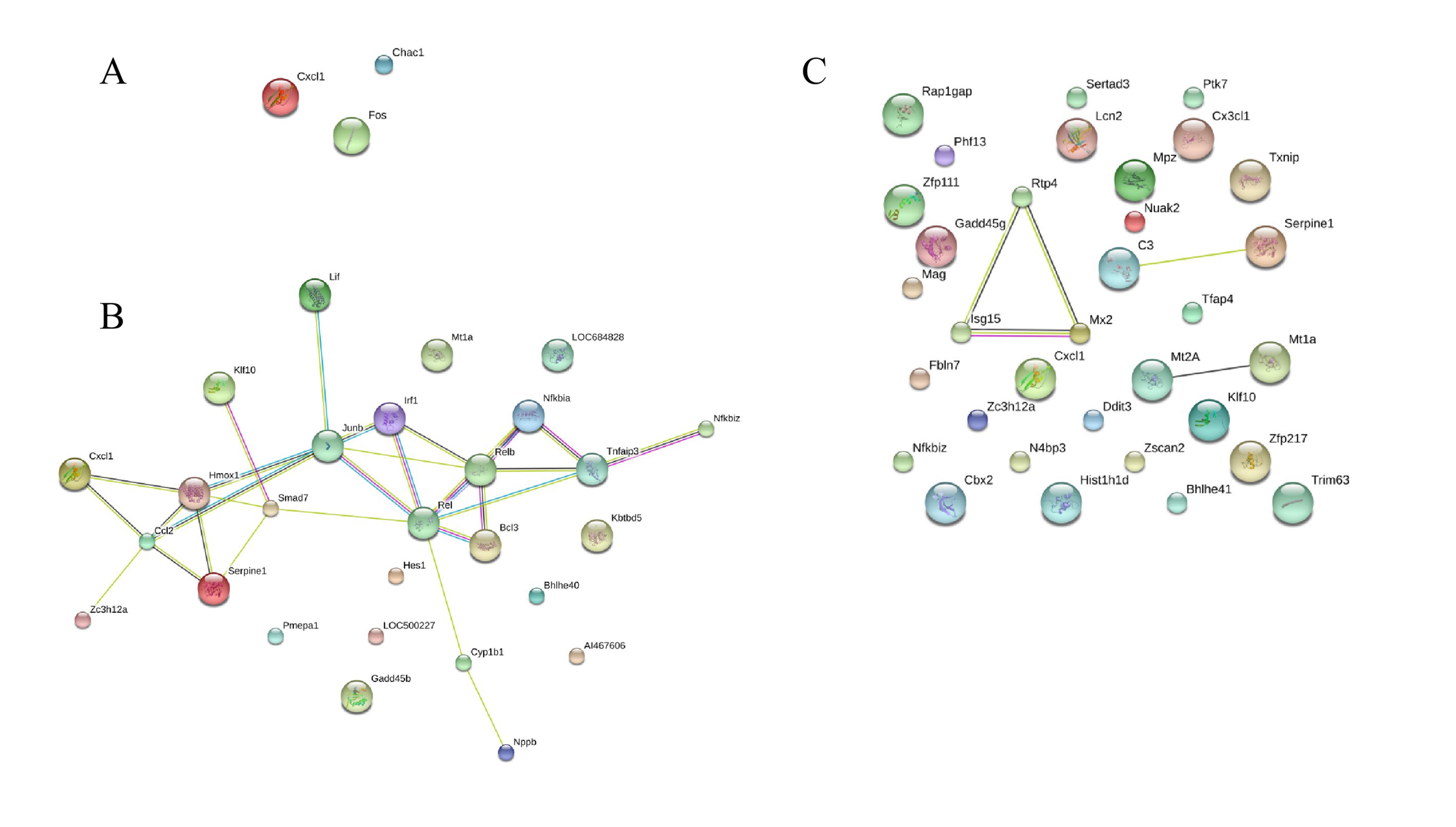

Supplement: S5 Fig — (A) The group of exogenous GDNF for 0.5 h. (B) The group of exogenous GDNF for 1 h. (C) The group of exogenous GDNF for 24 h. (TIF) [file pone.0289071.s005.tif]

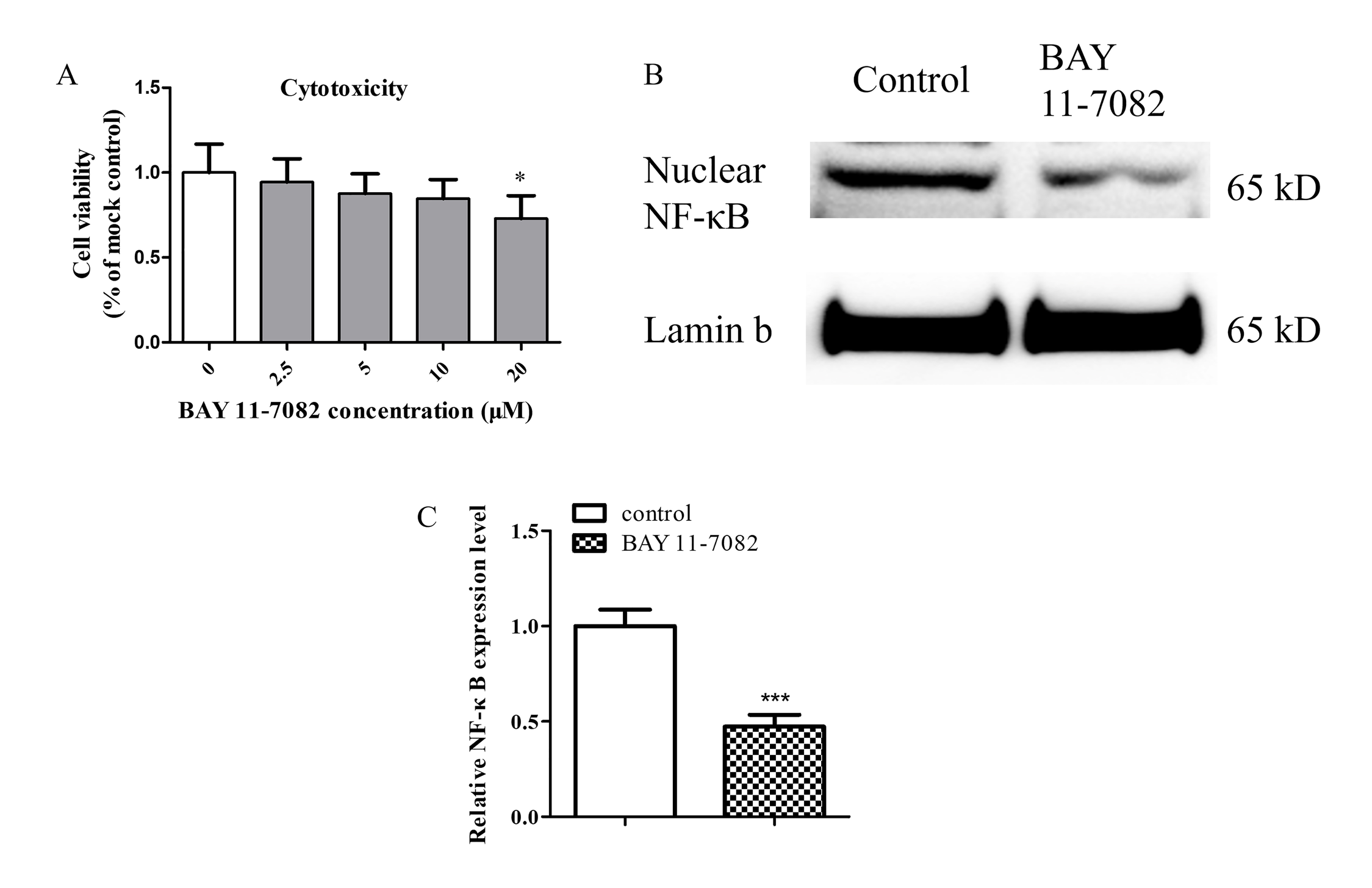

Supplement: S6 Fig — (A) Cytotoxicity of BAY 11–7082 to C6 cells. (B) Western blot showed that the expression level of CXCL1 in the BAY 11–7082 treated group and the control group. (C) The statistical results of (A). (*P<0.05, ***P<0.001). (TIF) [file pone.0289071.s006.tif]

Figure 4D

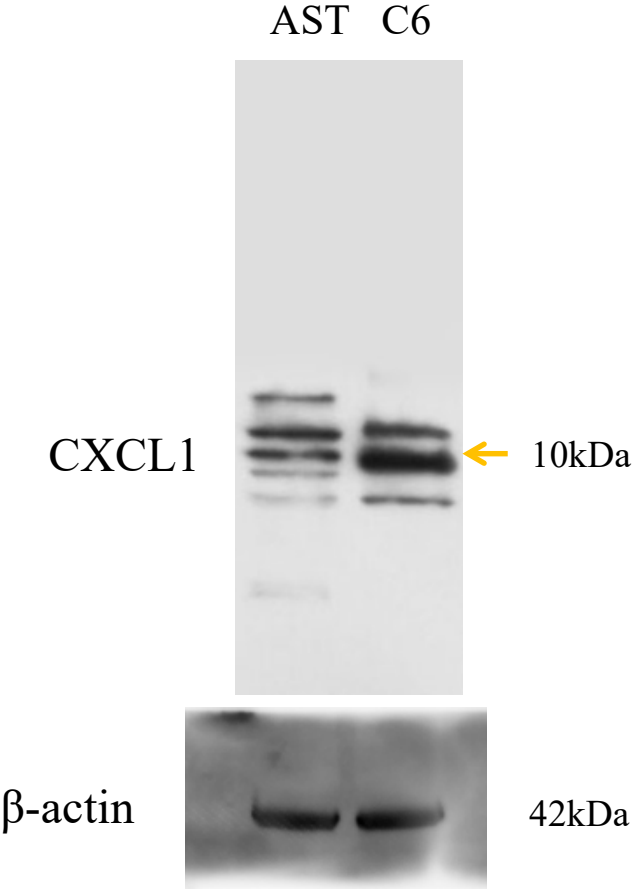

Figure 5A

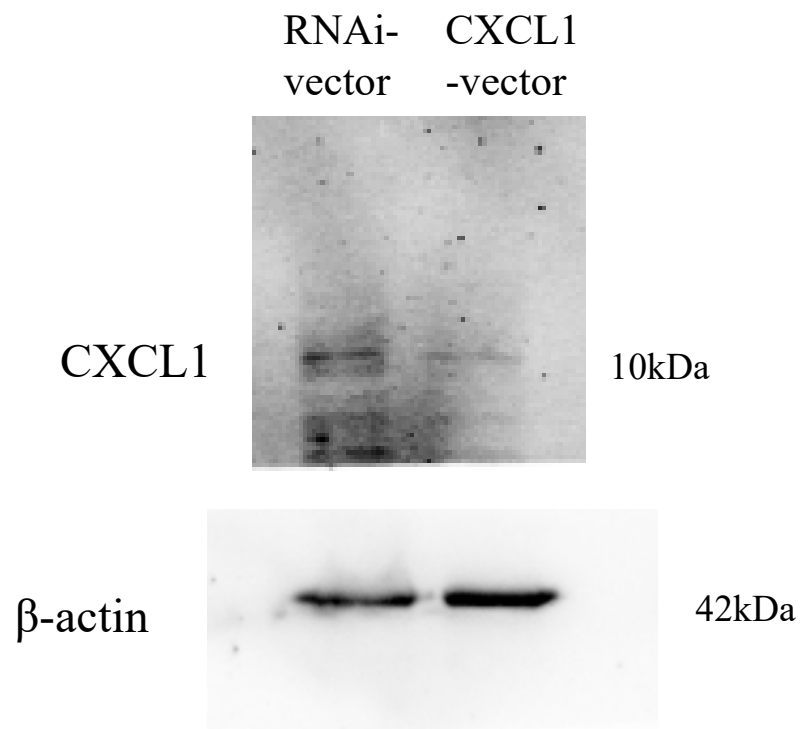

Figure 7C

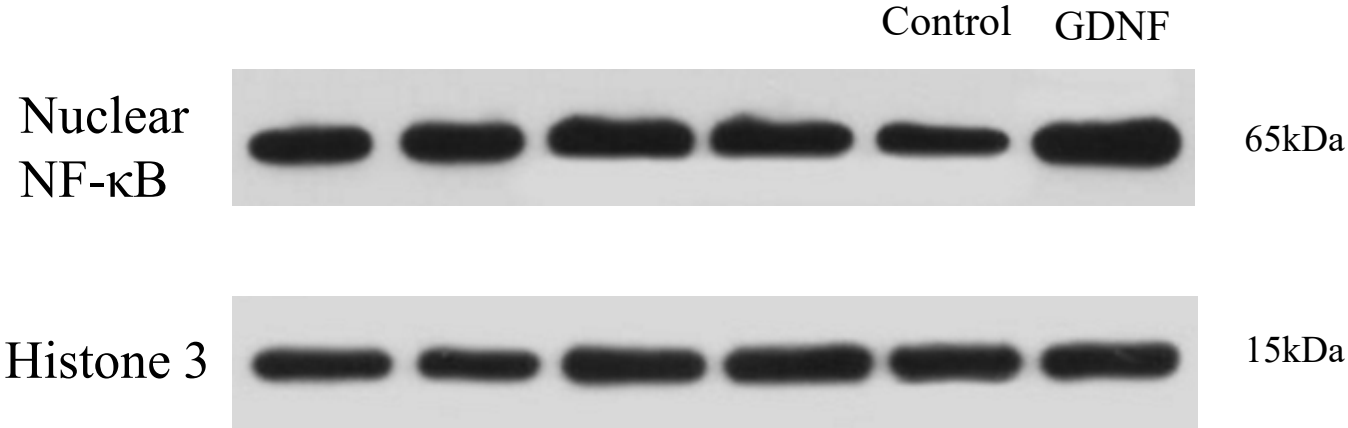

Figure 8A

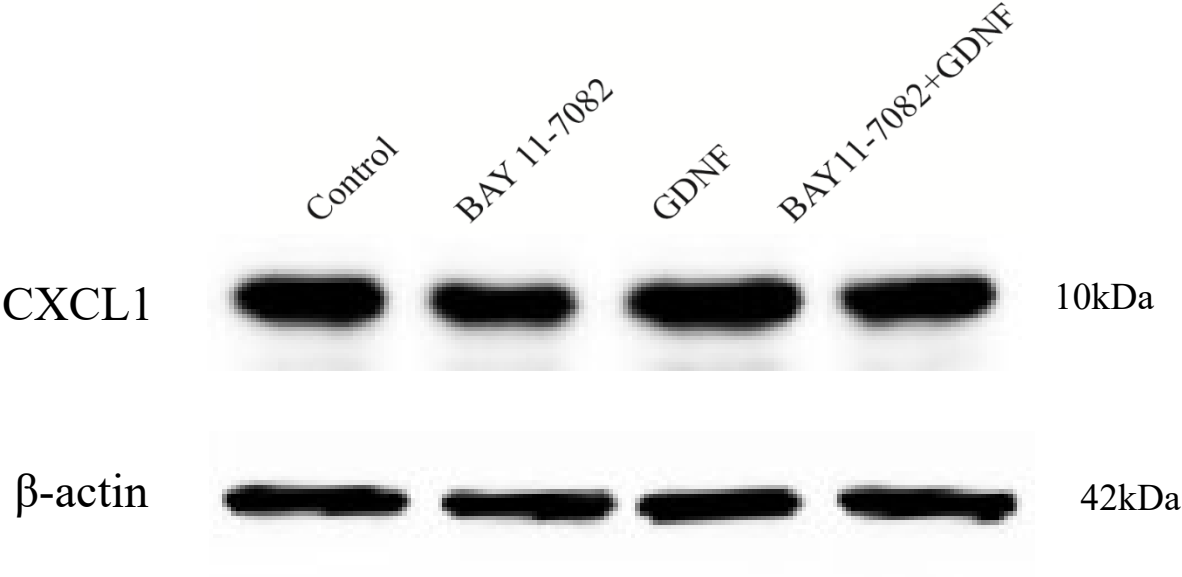

## Supplemental 6B

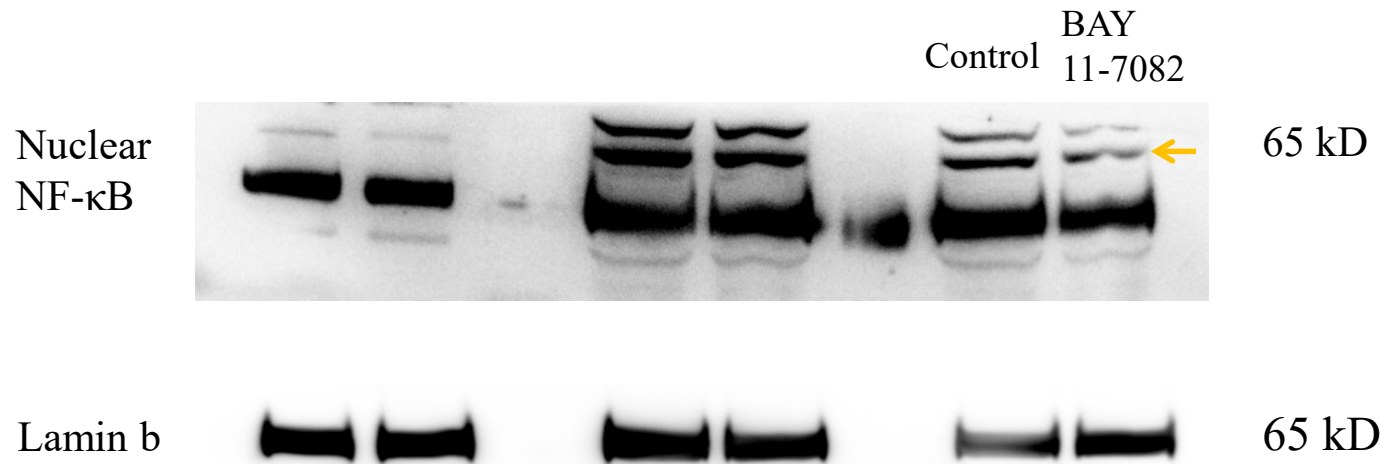

Supplement: S1 Raw images — (PDF) [file pone.0289071.s007.pdf]
